# Pathogen identification, biological characteristics and control agent screening of China Xinjiang white clover anthracnose

**DOI:** 10.3389/fpls.2026.1880984

**Published:** 2026-07-15

**Authors:** Yu Yuan Li, Xu Ke Zhang, Lei Shan, Saimi Wusiman, Jun Lin, Xuan Li, Ben Zhong Fu, Li Li Wang

**Affiliations:** 1 Department of Plant Pathology, College of Agronomy, Xinjiang Agricultural University, Key Laboratory of Prevention and Control of Invasive Alien Species in Agriculture & Forestry of the North-western Desert Oasis, Ministry of Agriculture and Rural Affairs, Key Laboratory of the Pest Monitoring and Safety Control of Crops and Forests, Urumqi, China; 2 Center for Grassland Biological Disaster Prevention and Control of Xinjiang Uygur Autonomous Region, Urumqi, Xinjiang, China

**Keywords:** anthracnose, biological characteristics, *Colletotrichum destructivum*, fungicide toxicity, pathogen identification, *Trifolium repens*

## Abstract

White clover is a high-quality forage and turfgrass. In 2023, a severe anthracnose was observed on white clover in Urumqi and Altay, Xinjiang, China. The pathogen was identified as *Colletotrichum destructivum* based on morphology and phylogeny. Biological characteristic assays revealed that the optimum temperature for mycelial growth was 25 °C, with an optimal pH range of 7.0 – 10.0. The most favorable media for growth were PDA, PSA and CA. Soluble starch and inulin served as the optimal carbon sources, while peptone was the most effective nitrogen source. Mycelial growth occurred under three tested light conditions (24 hours of light, 24 hours of darkness, and 12 hours of light/darkness), and the lethal temperature for mycelia was 54 °C for 10 minutes. The optimal conditions for sporulation were 20 °C, pH 6.0, under continuous darkness (24 h), with PDA as the medium, lactose as the carbon source, and peptone as the nitrogen source. *In vitro* toxicity tests of ten fungicides against *C. destructivum* demonstrated that 20% Imazalil was the most effective with EC_50_ = 0.1347 mg/L and followed by 430 g/L Tebuconazole with EC_50_ = 0.2239 mg/L. This study identifies the pathogen and biological features of white clover anthracnose in Xinjiang and identifies highly effective fungicides for its management, providing a theoretical basis for field control strategies.

## 1 Introduction

White clover (*Trifolium repens* L.), a perennial herbaceous plant belonging to the genus *Trifolium* within the family Fabaceae, is valued for its significant ecological and economic contributions. As a natural forage, its extensive root system and symbiotic rhizobia play a crucial role in maintaining soil moisture and fertility while enhancing soil aggregate structure. When utilized as fresh forage or hay, *T. repens* is characterized by high crude protein and mineral content in its dry matter. Its superior palatability, digestibility, and nutritional profile—surpassing those of red clover (*Trifolium pratense*) and alfalfa (*Medicago sativa*)—establish it as a high-yielding, premium legume forage (Mao, 2015). Furthermore, its robust adaptability to acidic or alkaline soils, alongside significant cold, heat, and drought tolerance, makes it a pivotal species for ecological restoration. Beyond its agricultural value, *T. repens* is widely employed in urban landscaping, including city greening, garden lawns and public green spaces (Shen et al., 2011; Abberton and Marshall, 2005).

However, the cultivation and application of white clover are hindered by various diseases, among which root rot and anthracnose pose the most catastrophic risks. Root rot in clover is typically caused by a complex of highly pathogenic *Fusarium species*, including *Fusarium avenaceum*, *F. equiseti*, *F. moniliforme*, *F. oxysporum*, *F. redolens*, *F. roseum*, and *F. solani* (Leath and Kendall, 1978; Mills, 1985; You et al., 2005; Zhang et al., 2024). Additionally, other pathogens such as *Verticillium* sp. (Milton and Isaac, 1976 and *Phytophthora megasperma* (Williams and Pascoe, 1994) have been implicated in the decline of clover stands.

Anthracnose in clover is associated with a diverse range of fungal pathogens (Talhinhas and Baroncelli, 2023). These include *Colletotrichum destructivum*, reported in the United States, the Netherlands, and China (Latunde-Dada et al., 1997; Damm et al., 2014; Xue et al., 2018; Tuluhong et al., 2024); *C. trifolii* in the United States and China (Nelson and Campbell, 1993; Damm et al., 2013); *C. lini* in the United States, Germany, and New Zealand; and *C. utrechtense*, currently documented only in the Netherlands (Damm et al., 2014). Furthermore, *C. truncatum* has been identified affecting clover specifically in China. Tuluhong et al. (2024) observed that *C. destructivum* isolates from Heilongjiang Province, China, exhibited higher pathogenicity on white clover than *C. truncatum*, with both species showing high sensitivity to tebuconazole in fungicide trials. To date, clover anthracnose in China has primarily been reported in the southwestern and eastern regions (Xue et al., 2018; Tuluhong et al., 2024). There remains a significant knowledge gap regarding the occurrence and characteristics of white clover anthracnose in Xinjiang.

In August 2023, severe outbreaks of anthracnose were observed on white clover in Urumqi and Altay, Xinjiang Uyghur Autonomous Region, China. The disease causes damage across all developmental stages, from seedlings to mature plants, affecting roots, stems, leaves, and flowers, leading to symptoms such as leaf blight, flower rot, and root rot. Severe infestation can lead to the death of the entire plant, resulting in premature thinning, decline, and eventual degradation of the turf stand. The objectives of this study were to: (1) identify the pathogenic species responsible for white clover anthracnose in this region; (2) characterize its biological properties; and (3) screen for highly effective, low-toxicity fungicides *in vitro*. This research aims to provide a scientific and theoretical foundation for the cultivation, management, and green integrated pest management (IPM) of white clover in Xinjiang.

## 2 Materials and methods

### 2.1 Isolation of fungal strains and morphological characterization

In 2023, we conducted a survey on root rot diseases of grassland plants in 13 counties and cities across Xinjiang, employing the random field sampling method for data collection. Ten white clover plants exhibiting distinct symptoms of basal stem necrosis, wilting, and leaf spotting were collected from Urumqi (43°33′N, 87°13′E) and Altay (47°29′N, 88°52′E) in the Xinjiang Uyghur Autonomous Region, China. From 10 diseased plants collected in the field, cultures were isolated from the symptomatic stem bases and roots. Pathogen isolation was performed using conventional tissue separation methods. Small tissue segments (3 mm × 3 mm) were excised from the margins of diseased root tissues. These segments were surface disinfected sequentially with 75% ethanol for 30 s and 2% NaClO for 3 min, followed by three rinses with sterile distilled water. The disinfected tissues were inoculated onto Potato Dextrose Agar (PDA) plates and incubated at 25 °C for 3–4 d. Newly grown hyphae from the colony edges were subcultured three times to ensure purity. Pure cultures were stored in 20% glycerol at -40 °C.

Isolates were activated on PDA at 25 °C for 5–7 d to observe colony characteristics. Conidiogenous structures and conidia were observed using a microscope (DS-Ri2), and the dimensions of 30 conidia (n = 30) were measured. Morphological identification—including the size and shape of setae, conidia, and appressoria—was conducted following the criteria established by Talhinhas (Talhinhas and Baroncelli, 2023).

### 2.2 Molecular biology identification

Genomic DNA was extracted from fresh mycelia of representative strains CS22 using a Plant Genomic DNA Kit (Tiangen Biotech, Beijing, China). Five loci, including the internal transcribed spacer (ITS), beta-tubulin (TUB2), glyceraldehyde-3-phosphate dehydrogenase (GAPDH), chitin synthase 1 (CHS-1), and actin (ACT), were amplified via PCR (**Table 1**).

**Table 1:** PCR primer information and reaction conditions.

Gene	Primer	Sequences (5’–3’)	Annealing temperature (°C)	Ref.
ITS	ITS1	TCCGTAGGTGAACCTGCGG	56	(White et al., 1990)
	ITS4	TCCTCCGCTTATTGATATGC		
GAPDH	GDF1	GCCGTCAACGACCCCTTCATTGA	60	(Guerber et al., 2003)
	GDR1	GGGTGGAGTCGTACTTGAGCATGT		
CHS-1	CHS-79F	TGGGGCAAGGATGCTTGGAAGAAG	55	(Myllys et al., 2002)
	CHS-345R	TGGAAGAACCATCTGTGAGAGTTG		
TUB2	BT2A	GGTAACCAAATCGGTGCTGCTTTC	58	(Glass and Donaldson, 1995)
	BT2B	ACCCTCAGTGTAGTGACCCTTGGC		
ACT	512F	ATGTGCAAGGCCGGTTTCGC	58	(Carbone and Kohn, 1999)

The 25 µL PCR reaction mixture contained: 2 µL DNA template, 1 µL of each primer (10 µmol/L), 12.5 µL 2 × PCR Mastermix (40 U Taq polymerase/mL), and 8.5 µL ddH_2_O. Thermal cycling conditions were: initial denaturation at 94 °C for 5 min; 35 cycles of denaturation at 94 °C for 30 s, annealing (**Table 1**) for 30 s, and extension at 72 °C for 1 min; with a final extension at 72 °C for 10 min. PCR products were analyzed via 2% agarose gel electrophoresis. Amplicons with single, clear bands were sequenced by Sangon Biotech (Shanghai, China).

Sequences were analyzed using BLAST against the NCBI database. Multi-locus sequences were aligned and concatenated using PhyloSuite v1.2.1. A phylogenetic tree was constructed using the Maximum Likelihood (ML) method (Nguyen et al., 2015) in raxmlGUI 2.0 with 1,000 bootstrap replicates.

### 2.3 Pathogenicity assays

The epidermis of 60-day-old white clover roots was slightly wounded using a sterile needle. The roots were then immersed in a spore suspension (1 × 10^6^ spores/mL) for 10–15 min. The treated plants were transplanted into pots, and 10 mL of the same suspension was applied as a soil drench around the root zone. Control plants were treated with sterile water. Three replicates (pots) were used per treatment, with three plants per pot. Disease incidence was recorded 30 d post-inoculation (Fang et al., 2019).

### 2.4 Biological characteristics determination

Colony Growth Assays. Mycelial plugs (5 mm diameter bacterial pellets were obtained from the edges of colonies cultured for 5 d) were inoculated onto 15 mL various media and incubated to assess growth under different conditions. Temperature: 5, 10, 15, 20, 25, 30, and 35 °C. pH: 3.0 – 11.0 (adjusted with 1 mol/L HCl or NaOH). Light regimes: 24 h light, 12 h light/dark cycle, and 24 h dark. Media: PDA, PSA, PCA, CA, OMA, CMA, WA, and Czapek (**Table 2**). Carbon/Nitrogen sources: Tested using Czapek agar as a base medium, replacing the carbon/nitrogen source with equal masses of test compounds. Colony diameter was measured using the cross-intersection method after 5 d at 25 °C.

**Table 2:** Compositions and formulations of the culture media used in this study.

Medium	Ingredients (per 1000 mL of distilled water)
Potato Eextrose Agar (PDA)	200 g Potato infusion, 20 g Dextrose, 17 g Agar
Potato Carrot Agar (PCA)	20 g Potato infusion, 20 g Carrot infusion, 17 g Agar
Potato Sucrose Agar (PSA)	200 g Potato infusion, 20 g Sucrose, 17 g Agar
Oatmeal Agar (OMA)	30 g Oatmeal, 17 g Agar
Cornmeal Agar (CMA)	30 g Cornmeal, 17 g Agar
Water Agar (WA)	17 g Agar
Carrot Agar (CA)	200 g Carrot infusion, 17 g Agar
Czapek-Dox Agar (Czapek)	2 g NaNO_3_, 1 g K_2_HPO_4_, 1 g KCl, 0.5 g MgSO_4_·7H_2_O, 0.01 g FeSO_4_·7H_2_O, 30 g Sucrose, 20 g Agar

Sporulation Assays. After 10 d of incubation on the aforementioned media, 10 mL of sterile water was added to each plate to prepare a spore suspension. Spore concentration was determined using a hemocytometer.

Lethal Temperature. Mycelial plugs in 2 mL sterile water were subjected to a temperature gradient (40 – 60 °C at 5 °C intervals) for 10 min. Once the range was identified, 1 °C increments were used to pinpoint the exact lethal temperature.

Each of the above treatments was performed with three replicates. Data were statistically analyzed via ANOVA and LSD tests (*P < 0.05*) using Excel 2019 and SPSS 23.0.

### 2.5 *In vitro* fungicide screening

The inhibitory effect of various fungicides (**Table 3**) was evaluated using the mycelial growth rate method. Stock solutions (1 × 10^5^ mg/L or 1 × 10^4^ mg/L for specific agents) were prepared in sterile water and stored at 4 °C. Five concentrations were set for each fungicide to prepare PDA plates. Mycelial plugs (5 mm) were inoculated and incubated at 25 °C for 5 d. The inhibition rate was calculated by comparing treated colony diameters with the control. EC_50_ values and toxicity regression equations were calculated using DPS 7.05 software.

**Table 3:** Fungicides for testing and information.

Fungicide type	Active ingredient (concentration)	Formulation	Manufacturer
Botanical	80% Ethylicin	EC	Shandong Guihe Biotechnology Co., Ltd.
	20% Eugenol	EW	Jiangsu Jianpai Agrochemical Co., Ltd.
Biological	10% Polyoxin	WP	Kaken Pharmaceutical Co., Ltd. (Japan)
	6% Kasugamycin	AS	Shanxi Xinyuan Huakang Chemical Co., Ltd.
	6% Ningnanmycin	AS	Deqiang Biology Co., Ltd.
	3% Zhongshengmycin	WP	Shenzhen Noposion Agrochemical Co., Ltd.
Chemical	50% Carbendazim	WP	Sichuan Runer Technology Co., Ltd.
	20% Imazalil	EW	Rotam Chemical Co., Ltd. (Jiangsu)
	430 g/L Tebuconazole	SC	FMC Corporation (Suzhou)
	80% Mancozeb	WP	Sichuan Guoguang Agrochemical Co., Ltd.


$$\text{Suppression rate}=\frac{\text{Diameter of Control}-\text{diameter of treated}}{\text{Diameter of Control}-\text{diameter of the plug}}\times 100\%$$

## 3 Results

### 3.1 Field symptoms

**Figure 1:**
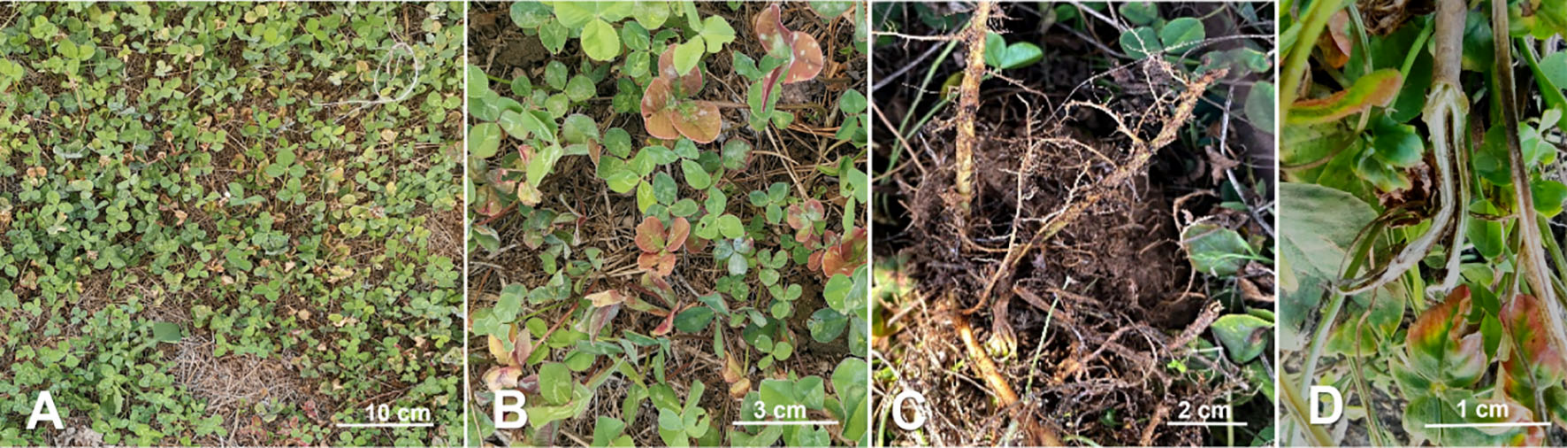
Symptoms of white clover anthracnose in Urumqi. **(A)** Aboveground symptoms. **(B)** The leaves have turned brown. **(C)** Root necrosis. **(D)** Necrosis within the root.

Anthracnose of white clover primarily affects the stems of seedlings and the stems, leaves, petioles, and roots of mature plants; it also infects inflorescences and seeds. Infected plants often develop leaf spots that are initially small and black, rapidly expanding into large, irregular lesions that frequently encompass entire leaflets. Stem lesions appear as elongated, sunken brown streaks. Upon infection of petioles and seedlings, symptoms begin as water-soaked spots that develop longitudinally into brown necrotic streaks. Severe infections at the stem base or root crown can lead to whole-plant mortality (**Figure 1**).

### 3.2 Morphological identification

**Figure 2:**
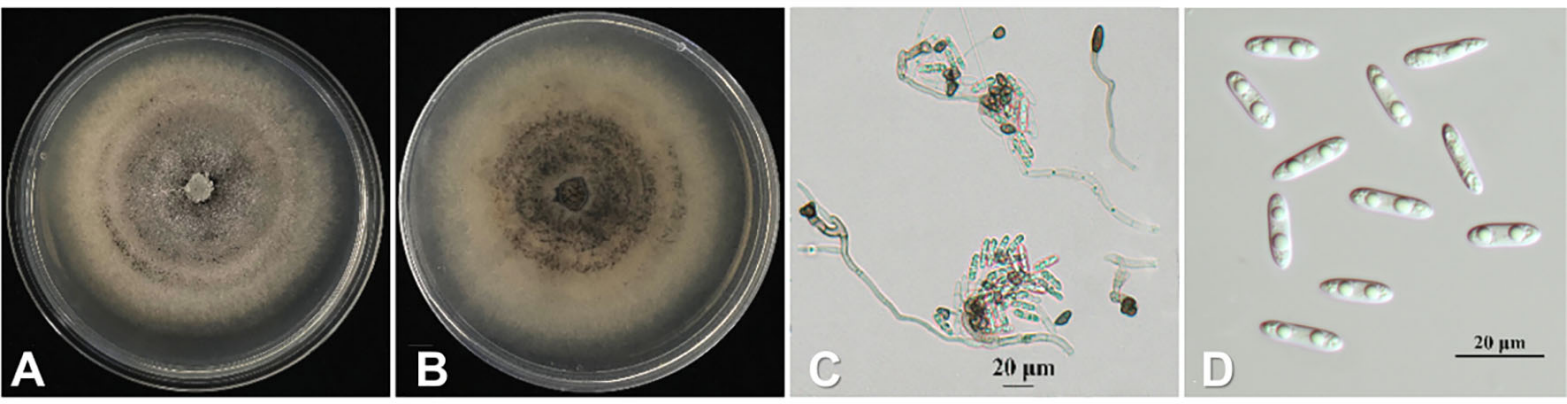
Colony characteristics and microscopic morphology of strain CS22 (5 d on PDA). **(A)** Colony surface. **(B)** Colony reverse. **(C)** Appressoria formed by hyphae. **(D)** Conidia.

A total of 67 *Colletotrichum* isolates were obtained from ten diseased white clover plants collected in Urumqi, Xinjiang. These isolates exhibited uniform morphological characteristics; therefore, a representative strain, CS22, was selected for detailed characterization. On Potato Dextrose Agar (PDA), CS22 colonies initially appeared white with sparse, highly branched aerial hyphae. Over time, the colonies turned brown to dark brown, forming punctate black acervuli (40 – 310 μm in diameter). Setae were dark brown, straight or slightly curved, and measured 60 – 170 μm in length. Conidiophores were hyaline and cylindrical, measuring 9.52 – 17.70 × 3.6 – 5.26 μm. Conidia were hyaline and single-celled, ranging from cylindrical with rounded ends to slightly tapered at the tips, and typically contained two central oil droplets 13.26 – 22.70 × 3.02 – 4.26 μm. Appressoria were thick-walled, brownish-brown, smooth, and single-celled; they were primarily spherical but occasionally clavate 6.22 – 9.20 × 5.68 – 7.32 µm. Based on these morphological traits, the isolate was tentatively identified as *Colletotrichum destructivum* (**Figure 2**).

### 3.3 Molecular phylogenetic identification

**Figure 3:**
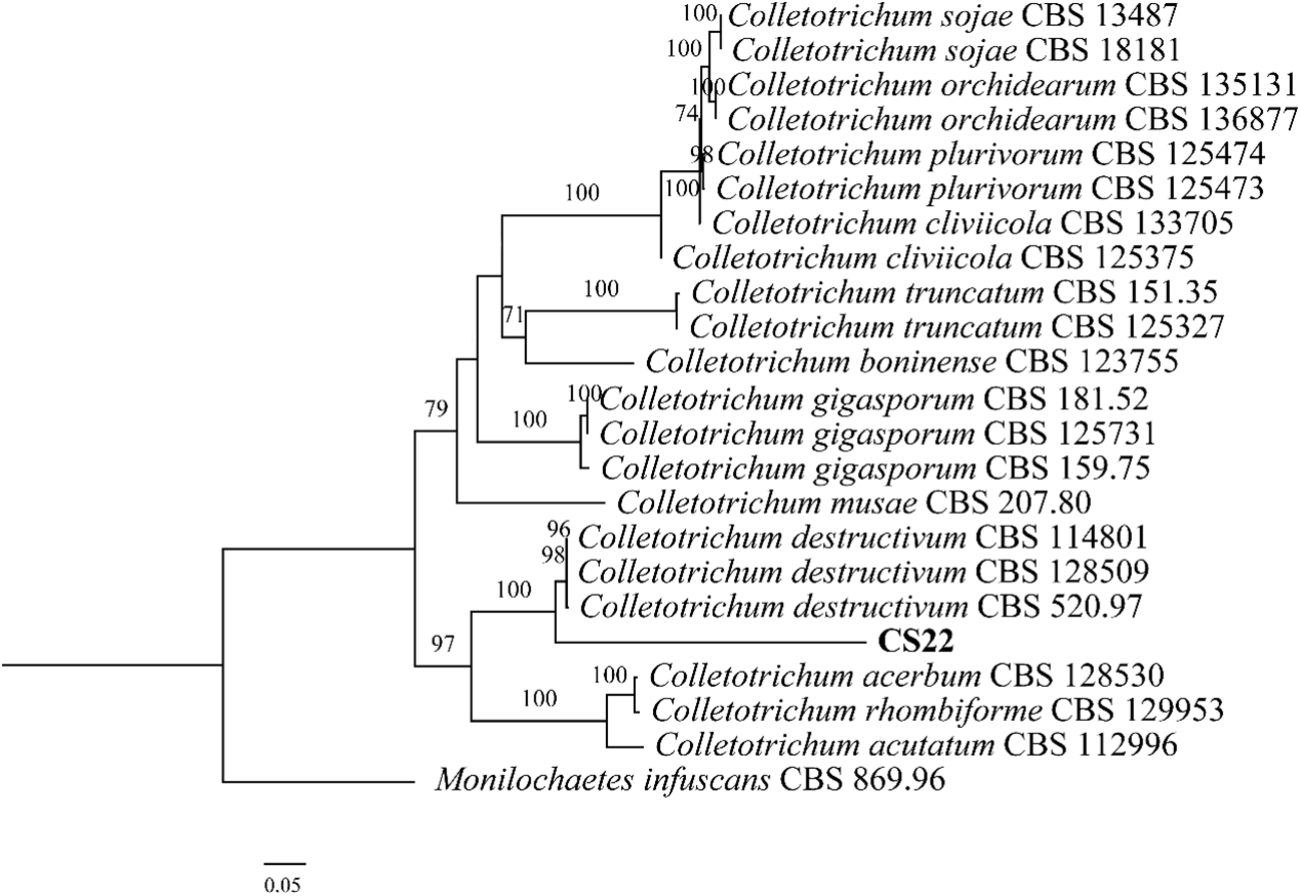
Strain CS22 constructed ML phylogenetic tree based on ITS, *ACT, CHS-1, TUB2* and *GAPDH* multi-gene sequences. Bootstrap values (greater than 40%) based on 1,000 replicates are shown at branch nodes. The scale bar represents 0.05 substitutions per nucleotide position. *Monilochaetes infuscans* sequences were used as an outgroup.Branches with less than 70% are not indicated.

PCR amplification of strain CS22 using primers for ITS, *ACT*, *CHS-1*, *TUB2* and *GAPDH* yielded products of 565, 242, 271, 609 and 168 bp, respectively. The sequences were deposited in GenBank with accession numbers PP486307 (ITS), PP488373 (*ACT*), PP488374 (*CHS-1*), PP488375 (*TUB2*), and PP488372 (*GAPDH*). A multi-locus phylogenetic tree was constructed using concatenated sequences, with *Monilochaetes infuscans* CBS 869.96 as the outgroup (**Table 4**). Strain CS22 clustered with *C. destructivum* strains CBS128509, CBS520.97, and CBS114801 with 100% bootstrap support. Combined with morphological data, isolate CS22 was confirmed as *C. destructivum* (**Figure 3**).

**Table 4:** Information on strains of *Colletotrichum* spp. for constructing a phylogenetic tree.

Species	Strains	GenBank accession number				
		ITS	ACT	CHS-1	TUB2	GAPDH
*C. acutatum*	CBS 112996	JQ005776	JQ005839	JQ005797	JQ005860	JQ948677
*C. acerbum*	CBS 128530	JQ948459	JQ949780	JQ949120	JQ950110	JQ948790
*C. boninense*	CBS 123755	JQ005153	JQ005501	JQ005327	JQ005588	JQ005240
*C. cliviicola*	CBS 125375	MG600733	MG600939	MG600850	MG601000	MG600795
*C. cliviicola*	CBS 133705	MG600732	MG600938	MG600849	MG600999	MG600794
*C. destructivum*	CBS 128509	KM105214	KM105424	KM105284	KM105494	KM105569
*C. destructivum*	CBS 520.97	KM105217	KM105427	KM105287	KM105497	KM105572
*C. destructivum*	CBS 114801	KM105219	KM105429	KM105289	KM105499	KM105574
*C. gigasporum*	CBS 159.75	KF687726	KF687783	KF687776	KF687884	KF687839
*C. gigasporum*	CBS 125731	KF687727	KF687794	KF687771	KF687879	KF687837
*C. gigasporum*	CBS 181.52	KF687734	KF687799	KF687775	KF687885	KF687838
*C. musae*	CBS 20780	KC566790	KC566936	KC566357	KC566212	KC566644
*C. orchidearum*	CBS 135131	MG600738	MG600944	MG600855	MG601005	MG600800
*C. orchidearum*	CBS 136877	MG600739	MG600945	MG600856	MG601006	MG600801
*C. plurivorum*	CBS 125473	MG600717.	MG600924	MG600840	MG600984	MG600780
*C. plurivorum*	CBS 125474	MG600718	MG600925	MG60084	MG600985	MG600781
*C. rhombiforme*	CBS 129953	JQ948457	JQ949778	JQ949118	JQ950108	JQ948788
*C. sojae*	CBS 13487	MG600752	MG600957	MG600863	MG601019	MG600813
*C. sojae*	CBS 18181	MG600753	MG600958	MG600864	MG601020	MG600814
*C. truncatum*	CBS 125327	GU227887	GU227985	GU228377	GU228181	GU228279
*C. truncatum*	CBS 151.35	GU227862	GU227960	GU228352	GU228156	GU228254
*Monilochaetes infuscans*	CBS 869.96	JQ005780	JQ005843	JQ005801	JQ005864	JX546612

### 3.4 Pathogenicity assay

**Figure 4:**
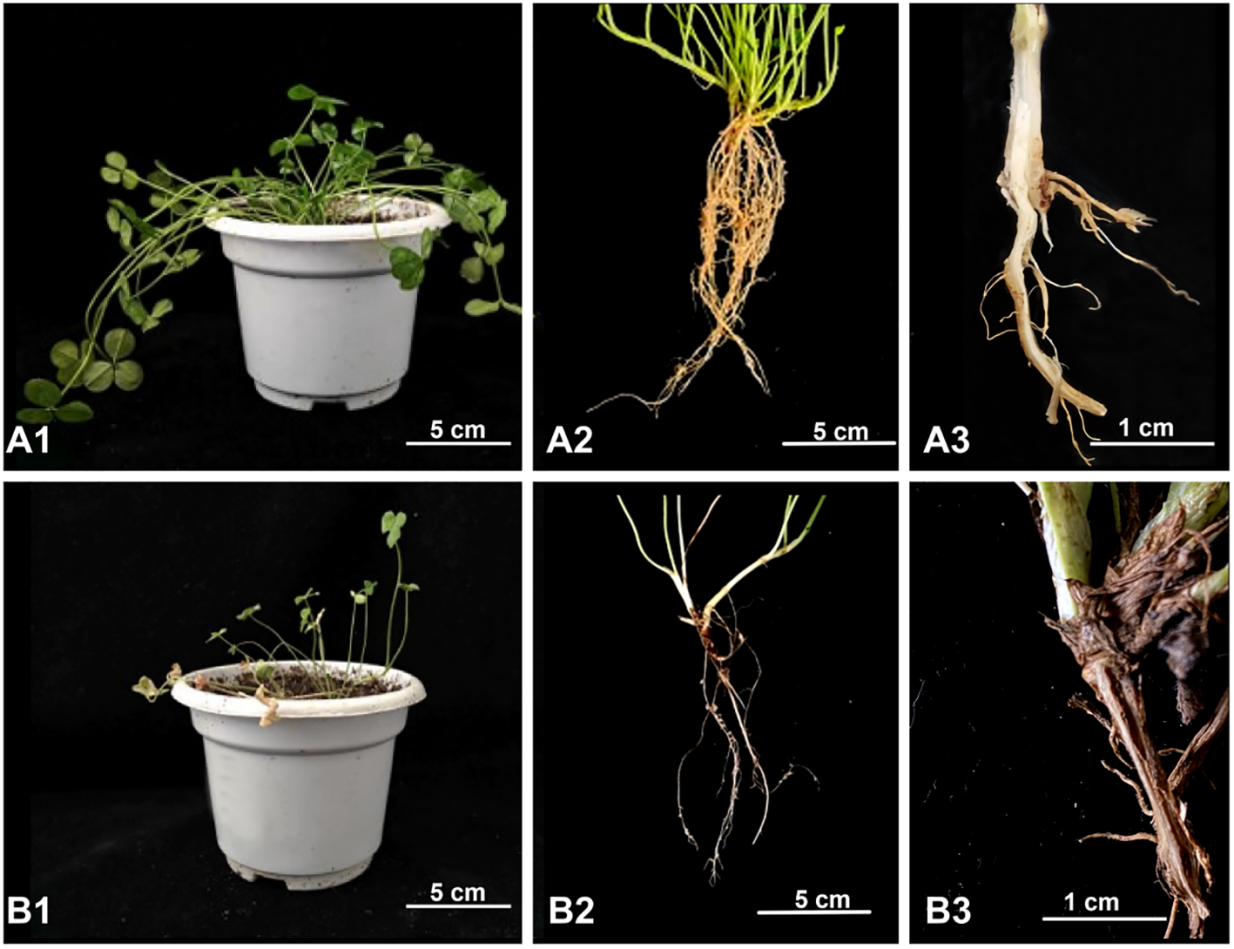
Pathogenicity assay of *Colletotrichum destructivum* (CS22) on white clover at 30 days post-inoculation (dpi). **(A1)** treated with sterile water (control). **(A2)** Root in the control group. **(A3)** longitudinal section of root. **(B1)** inoculated with the pathogen. **(B2)** Root symptoms. **(B3)** treated root with longitudinal section. Scale bar marked on each panel.

The pathogenicity of strain CS22 was evaluated using a root-drenching inoculation method. Two weeks post-inoculation (wpi) with the spore suspension, treated plants exhibited stunted growth and initial leaf wilting. Over time, chlorosis developed at the leaf tips and margins. By 30 days post-inoculation (dpi), the plants were significantly dwarfed, drooping, and sparse with yellowing foliage. Brown to black necrotic lesions appeared at the stem bases and roots. Additionally, infected plants showed shortened primary roots and a reduced number of fibrous roots, with brown necrotic streaks. These symptoms were consistent with field observations of white clover root rot. Re-isolation from symptomatic root tissues yielded isolates identical to the original inoculum, confirming Koch’s postulates. In the sterile water control (CK), plants at 30 dpi exhibited longer stolons, dense foliage, and vigorous growth without chlorosis. The roots remained naturally pale yellow, well-developed, and rich in fibrous roots, with no evidence of necrosis (**Figure 4**).

### 3.5 Biological characteristics

#### 3.5.1 Temperature

Mycelial growth and sporulation occurred between 5 and 35 °C. The optimal temperature for mycelial growth was 25 °C (5.67 cm diameter at 5 d), while the optimal temperature for sporulation was 20 °C (5.03 × 10^6^ spores/mL at 10 d). Growth and sporulation significantly decreased below 15 °C or above 35 °C.

#### 3.5.2 pH

Mycelial growth showed no significant difference between pH 7.0 and 10.0, with the maximum diameter recorded at pH 10.0 (5.88 cm). Growth was not significantly retarded at pH 4.0 and 5.0. However, sporulation was significantly higher at pH 6.0 (0.93 × 10^6^ spores/mL) compared to other pH levels. (*P < 0.05*).

#### 3.5.3 Light

There was no significant difference in mycelial growth under the three tested light regimes (P > 0.05), although continuous light slightly favored growth (6.06 cm). In contrast, sporulation was highest under continuous darkness (0.57 × 10^6^ spores/mL).

#### 3.5.4 Media

Mycelial growth was most rapid on PDA, PSA, and CA (5.72, 5.56, and 5.33 cm, respectively), while growth was slowest on WA, CMA, and Czapek agar. PDA was the optimal medium for sporulation (14.28 × 10^6^ spores/mL), significantly exceeding other media (*P < 0.05*).

#### 3.5.5 Carbon and nitrogen sources

Soluble starch was the most effective carbon source for growth, followed by inulin and glucose. Lactose was the optimal carbon source for sporulation (13.38 × 10^6^ spores/mL), while other sources induced negligible sporulation. Peptone was the optimal nitrogen source for both mycelial growth (5.92 cm) and sporulation (5.03 × 10^6^ spores/mL).

#### 3.5.6 Lethal temperature

The mycelia could survive 10-minute water bath treatments at 40, 45, and 50 °C but failed to grow at 55 °C. Further refinement identified 54 °C as the precise lethal temperature for *C. destructivum* (**Table 5**).

**Table 5:** Effects of different culture conditions on mycelial growth and sporulation of *C. destructivum*.

Test	Culture condition	Colony diameter (cm)	Spore number (× 10^6^ spore/mL)
Temperature (°C)	5	0.82 ± 0.05 d	0.00 ± 0.00 c
	10	1.09 ± 0.16 d	0.20 ± 0.05 c
	15	2.12 ± 0.30 c	0.03 ± 0.01 c
	20	4.42 ± 0.20 b	5.03 ± 1.68 a
	25	5.67 ± 0.38 a	0.30 ± 0.05 c
	30	4.76 ± 0.51 b	3.43 ± 0.83 b
	35	2.26 ± 0.18 c	0.38 ± 0.25 c
pH	4	4.31 ± 0.15 d	0.23 ± 0.15 d
	5	4.87 ± 0.23 c	0.43 ± 0.21 c
	6	5.25 ± 0.52 bc	0.93 ± 0.75 a
	7	5.86 ± 0.14 a	0.72 ± 0.33 b
	8	5.80 ± 0.05 a	0.10 ± 0.05 e
	9	5.48 ± 0.54 ab	0.20 ± 0.09 d
	10	5.88 ± 0.08 a	0.17 ± 0.08 de
Light regimes	24 h all light	6.06 ± 0.33 a	0.07 ± 0.03 b
	24 h all dark	5.58 ± 0.28 a	0.57 ± 0.08 a
	12 h light/dark	5.82 ± 0.11 a	0.07 ± 0.03 b
Culture Media	PDA	5.72 ± 0.44 a	14.28 ± 2.40 a
	PSA	5.56 ± 0.28 a	2.70 ± 1.99 b
	PCA	4.10 ± 0.16 c	0.17 ± 0.08 bc
	WA	2.38 ± 0.06 e	0.07 ± 0.05 c
	CMA	3.22 ± 0.17 d	0.30 ± 0.15 bc
	OMA	4.87 ± 0.08 b	0.37 ± 0.23 bc
	CA	5.33 ± 0.13 a	0.05 ± 0.05 c
	Czapek	3.32 ± 0.23 d	0.00 ± 0.00 c
Carbon sources	Glucose	3.85 ± 0.27 ab	0.02 ± 0.02 b
	Maltose	3.45 ± 0.13 abc	0.07 ± 0.04 b
	Lactose	3.19 ± 0.44 cd	13.38 ± 3.81 a
	Inulin	3.92 ± 0.33 a	0.03 ± 0.01 b
	D-fructose	2.66 ± 0.06 e	0.00 ± 0.00 b
	Mannitol	3.39 ± 0.04 bcd	0.06 ± 0.05 b
	Sorbitol	3.36 ± 0.23 bcd	0.10 ± 0.00 b
	Soluble starch	3.94 ± 0.51 a	0.28 ± 0.03 b
	Carbon-free control	2.87 ± 0.08 de	0.00 ± 0.00 b
Nitrogen Sources	Ammonium sulfate	3.57 ± 0.36 c	0.33 ± 0.17 c
	Ammonium chloride	3.58 ± 0.25 c	0.15 ± 0.05 c
	Peptone	5.92 ± 0.30 a	5.03 ± 1.00 a
	Beef extract	5.42 ± 0.05 b	1.58 ± 0.38 b
	Yeast extract	5.70 ± 0.24 ab	1.48 ± 0.86 b
	Glycine	3.31 ± 0.35 c	0.27 ± 0.12 c
	Phenylalanine	3.41 ± 0.23 c	0.53 ± 0.14 c
	Potassium nitrate	3.59 ± 0.31 c	0.05 ± 0.05 c
	Nitrogen-free control	3.61 ± 0.09 c	0.12 ± 0.08 c

### 3.6 *In vitro* toxicity of 10 fungicides

Toxicity tests (**Table 6**) revealed that 20% Imazalil and 430 g/L tebuconazole were the most potent inhibitors of mycelial growth, with EC_50_ values of 0.1347 and 0.2239 mg/L, respectively. 50% Carbendazim followed with an EC_50_ of 3.0629 mg/L. Polyoxin, Zhongshengmycin, and Ethylicin also showed moderate inhibitory effects. Ningnanmycin and Kasugamycin exhibited the lowest toxicity, with EC_50_ values exceeding 270 mg/L.

**Table 6:** Toxicity of 10 fungicides against C. destructivum in the laboratory.

Fungicide	Concentration (mg/L)	Toxicity regression equation	Correlation determination	EC_50_ (mg/L)
20% Eugenol	10, 20, 40, 80, 160	Y = 0.4937 + 2.1311 X	0.9898	130.1693
80% Ethylicin	5, 10, 20, 40, 80	Y = 1.9912 + 1.8755 X	0.9925	40.2014
6% Kasugamycin	50, 100, 200, 400, 800	Y = 0.1765 + 1.6737 X	0.9919	762.1172
10% Polyoxin	5, 10, 15, 20, 25	Y = 2.4651 + 1.8490 X	0.9486	23.4921
6% Ningnanmycin	25, 50, 100, 200, 400	Y = 0.4357 + 1.8660 X	0.9983	279.2429
3% Zhongshengmycin	6.25, 12.5, 25, 50, 100	Y = 1.8234 + 2.1096 X	0.9731	32.0464
20% Imazalil	0.125, 0.25, 0.5, 1, 2	Y = 6.1074 + 1.2719 X	0.9440	0.1347
430 g/L Tebuconazole	0.125, 0.25, 0.5, 1, 2	Y = 5.8211 + 1.2634 X	0.9639	0.2239
80% Mancozeb	9.375, 18.25, 37.5, 75, 150	Y = 3.1623 + 0.9572 X	0.9661	83.1431
50% Carbendazim	0.625, 1.25, 2.5, 5, 10	Y = 3.8220 + 2.4232 X	0.9791	3.0629

## 4 Discussion

From 2023 to 2024, our team conducted surveys and pathogen identification on root diseases of white clover in 13 counties and cities across Xinjiang, identifying cases of white clover anthracnose only in Altay and Urumqi. Under normal circumstances, the genus *Colletotrichum* comprises a globally distributed group of ascomycetous fungi capable of parasitizing a vast range of gymnosperms, angiosperms, monocots, and dicots. Ranked as the eighth most significant group of plant pathogenic fungi worldwide, *Colletotrichum* species cause devastating diseases, including leaf spot, leaf blight, flower rot, fruit rot, and root rot, leading to substantial economic losses and severely impacting plant development (Dean et al., 2012). In this study, isolation and culture of pathogenic bacteria from necrotic tissue at the rhizome site, through morphological characterization, multi-locus phylogenetic analysis, and pathogenicity assays, we identified *C. destructivum* as the causal agent of anthracnose on white clover (*T. repens*) in Xinjiang. Our findings align with previous reports of *C. destructivum* infecting white clover in Sichuan (Xue et al., 2018) and Heilongjiang (Tuluhong et al., 2024), China, suggesting a broad geographical distribution of this pathogen across diverse climatic regions of the country.

Our investigation into the biological characteristics of strain CS22 revealed that environmental factors significantly influence mycelial growth and sporulation. The preference of strain CS22 for PDA medium, peptone as a nitrogen source, and a temperature range of 25 – 30 °C for mycelial growth is consistent with findings reported by Ma et al. for *C. destructivum* on alfalfa (Ma et al., 2016) and Miao on *Cynanchum atratum* (Miao, 2017). However, while our results regarding soluble starch as the optimal carbon source, an optimal pH of 7 – 9, and adaptability to various light conditions concur with studies on *C. destructivum* isolated from *Echeveria* ‘Perle von Nürnberg’ (Dong, 2022) and grapes (Jiang, 2023), some discrepancies in reproductive requirements were noted.

Specifically, while the optimal medium (PDA), nitrogen source (peptone), and light regime (continuous darkness) for sporulation in our study matched the results of Ma et al (Ma et al., 2016)), the optimal carbon source, temperature, and pH requirements differed from those reported in literature (Ma et al., 2016; Miao, 2017). Furthermore, the lethal temperature for strain CS22 was determined to be 54 °C, which is higher than the 52 °C (Jiang, 2023) but lower than the 58 °C (Miao, 2017).

Regarding chemical control, all ten tested fungicides exhibited varying degrees of inhibitory activity against strain CS22. The chemical fungicides 20% imazalil and 430 g/L tebuconazole demonstrated significantly higher efficacy than the biological agents (kasugamycin, ningnanmycin, and polyoxin) and botanical extracts (eugenol and ethylicin). This disparity likely stems from differing modes of action. The high sensitivity of *C. destructivum* to imazalil observed here is consistent with its reported effectiveness against *Penicillium digitatum* (Smilanick et al., 1997) and *C. brevisporum* (Zhang et al., 2021). Similarly, the potent inhibitory effect of tebuconazole mirrors results observed for *C. truncatum*, *C. destructivum*, and *C. cereale* (Tuluhong et al., 2024; Wong and Midland, 2007). A concentration of 20% imazalil combined with 430 g/L tebuconazole requires further validation for both pot culture and field application efficacy.

Interestingly, 50% carbendazim, 10% polyoxin, 3% zhongshengmycin, and 80% ethylicin also displayed promising inhibitory effects. Our findings regarding ethylicin and zhongshengmycin are consistent with research Shi et al. (2021) on *C. gloeosporioides* in grapes, where these agents outperformed the conventional fungicide mancozeb. This suggests that these compounds possess broad-spectrum toxicity across different species within the *Colletotrichum* genus, offering viable alternatives for integrated disease management.

## 5 Conclusion

The high incidence of *Colletotrichum* infection on white clover in Xinjiang, China, severely compromises both landscape aesthetics and ecological functions, significantly reducing its economic value as a forage crop. This study identified *C. destructivum* as the primary pathogen responsible for white clover anthracnose in Xinjiang, China, through comprehensive morphological and multi-locus phylogenetic analyses. Our characterization of its biological properties defined the optimal environmental parameters for mycelial expansion and conidial production, while highlighting regional variations in the pathogen’s physiological requirements. *In vitro* fungicide screening identified 20% imazalil as the most potent inhibitor of the pathogen. These results provide a critical theoretical foundation for developing effective integrated control strategies to manage field white clover anthracnose.

## Data Availability

The datasets presented in this study can be found in online repositories. The names of the repository/repositories and accession number(s) can be found below: https://www.ncbi.nlm.nih.gov/, PP486307; https://www.ncbi.nlm.nih.gov/, PP488373; https://www.ncbi.nlm.nih.gov/, PP488374; https://www.ncbi.nlm.nih.gov/, PP488375; https://www.ncbi.nlm.nih.gov/, PP488372.
